# Interplay between structural parameters and reactivity of Zr_6_-based MOFs as artificial proteases†

**DOI:** 10.1039/d0sc02136a

**Published:** 2020-05-22

**Authors:** Alexandra Loosen, Francisco de Azambuja, Simon Smolders, Jens Moons, Charlotte Simms, Dirk De Vos, Tatjana N. Parac-Vogt

**Affiliations:** Department of Chemistry, KU Leuven Celestijnenlaan 200F Leuven Belgium tatjana.vogt@kuleuven.be; Department Microbial and Molecular Systems, KU Leuven Celestijnenlaan 200F Leuven Belgium

## Abstract

Structural parameters influencing the reactivity of metal–organic frameworks (MOF) are challenging to establish. However, understanding their effect is crucial to further develop their catalytic potential. Here, we uncovered a correlation between reaction kinetics and the morphological structure of MOF-nanozymes using the hydrolysis of a dipeptide under physiological pH as model reaction. Comparison of the activation parameters in the presence of NU-1000 with those observed with MOF-808 revealed the reaction outcome is largely governed by the Zr_6_ cluster. Additionally, its structural environment completely changes the energy profile of the hydrolysis step, resulting in a higher energy barrier Δ*G*^‡^ for NU-1000 due to a much larger Δ*S*^‡^ term. The reactivity of NU-1000 towards a hen egg white lysozyme protein under physiological pH was also evaluated, and the results pointed to a selective cleavage at only 3 peptide bonds. This showcases the potential of Zr-MOFs to be developed into heterogeneous catalysts for non-enzymatic but selective transformation of biomolecules, which are crucial for many modern applications in biotechnology and proteomics.

## Abstract 2

Peptide bond hydrolysis catalyzed by NU-1000 MOF revealed key structural features that influence the catalytic performance of Zr_6_ based MOFs.

## Graphical abstract

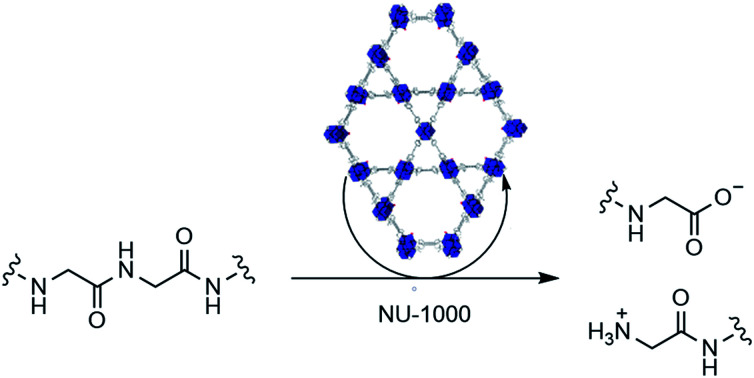

## Introduction

Selective hydrolytic cleavage of peptide bonds is key to many chemical and biological applications such as the study of protein function,^1^ the analysis of protein folding,^2^ proteomics,^3^ and the mapping of enzyme active sites.^4–6^ Proteolytic enzymes or peptidases commonly used for this purpose, *e.g.*, trypsin, are costly, and generally afford short peptide fragments which frequently results in partial protein sequence determination.^7^ Strategies combining multiple proteases to circumvent this issue have been described, but their cost is prohibitive for routine use.^7,8^ On the other hand, cheaper chemical agents such as the toxic and volatile cyanogen bromide are used in super-stoichiometric amounts and require harsh reaction conditions, leading to a number of undesired side reactions that hinder further analysis.^9^ In this context, hybrid nanomaterials with enzyme-like characteristics (nanozymes) recently emerged as an attractive alternative to develop artificial metalloproteases given their great stability, ready availability and potential recyclability that sharply decreases their economical cost.^10,11^ Despite this enormous potential, few examples of peptide bond hydrolysis have been reported so far,^12,13^ and deeper studies of different systems are required to use nanozyme peptidases in routine applications. To this end, we present here a detailed account of Zr_6_ based MOF NU-1000 peptidase activity towards short peptides and proteins, which provides smooth and selective (in case of protein) cleavage of the peptide bond under mild physiological conditions. In the course of this investigation, we observed an unexpected large entropy penalty for the hydrolysis step, which provides strong experimental evidence that the network structure plays a role as important as the composition in the catalytic activity of MOFs.

Several homogeneous artificial metalloproteases have been reported over the years, but heterogeneous catalysts for the selective peptide bond cleavage are still largely underdeveloped.^14^ During the past decade, we discovered and developed the unique hydrolytic activity of Zr(iv) substituted polyoxometalates (Zr^IV^-POMs) toward the selective cleavage of peptide bonds in short peptides and even proteins.^15–23^ However, purification and/or analysis of protein digest, as well as catalyst recyclability were hampered by the solubility of Zr^IV^-POMs in water, precluding any practical applications. Recently, we^12^ and others^13^ pioneered metal–organic frameworks (MOFs) as heterogeneous catalysts in the hydrolysis of peptides and proteins, inspired by their use in the hydrolysis of phosphoester bonds in nerve agents.^24–26^ Strikingly, peptide bond hydrolysis was faster than previously observed with Zr^IV^-POMs in both model peptides and protein substrates. Moreover, we could easily separate the catalyst from the crude reaction mixture through a simple centrifugation step. Motivated by this excellent prospect and the excellent mechanical,^27^ thermal,^28^ chemical,^29^ and hydrolytic stability in aqueous media and organic solvents,^28,30^ we decided to study other Zr-MOFs as heterogeneous artificial proteases to correlate their structure with reactivity and, in the case of protein hydrolysis, the with selectivity of cleavage.

**Fig. 1:**
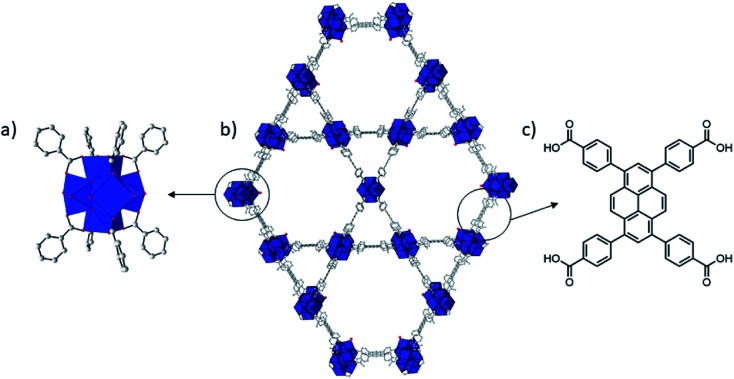
Structure of NU-1000. Zr = blue coordination polyhedron, O = red, C = gray. H atoms have been omitted for clarity. Zr_6_ cluster (a), NU-1000 (b), and TBAPy linker (c).

Among the known Zr(iv) based MOFs that could be developed as artificial proteases, NU-1000 attracted our attention due to its excellent activity towards the hydrolysis of phosphoester bonds in nerve agents^25^ and structural features that are quite distinct from our previously studied MOF-808.^12^ Zr-MOF NU-1000 consists of [Zr_6_(μ_3_–O)_4_(μ_3_–OH)_4_(H_2_O)_4_(OH)_4_]^8+^ octahedral nodes that are 8-connected by large 1,3,6,8-(*p*-benzoate)pyrene (TBAPy^4−^) linkers resulting in a csq net with hexagonal mesopores (31 Å) and triangular 12 Å micropores (Fig. 1).^31^ Compared to MOF-808, NU-1000 has higher connectivity, larger pores and higher BET-surface, which likely affects the reactivity of the catalyst towards substrates and can influence catalytic activity. In addition, the NU-1000 particles are larger and their sizes are more homogeneously distributed compared to MOF-808. Therefore, hydrolysis of peptides and proteins in the presence of NU-1000 could offer important insight into the MOF structural parameters that affect the catalytic cleavage. Such insights would be of great value not only for the development of MOF-based scarcely developed heterogeneous artificial proteases, but also for the development of more efficient MOF catalysts to other reactions of interest, enhancing the revolutionary prospect frequently attributed to MOFs.

## Results and discussion

### Hydrolysis of glycylglycine by NU-1000

**Fig. 2:**
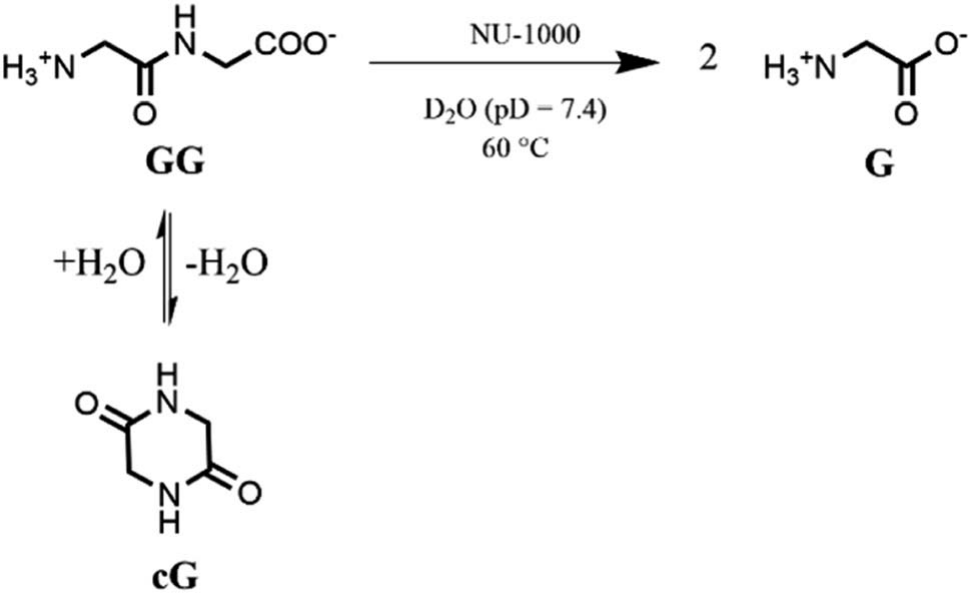
Hydrolysis and cyclization of GG to G and cG respectively in the presence of NU-1000.

**Fig. 3:**
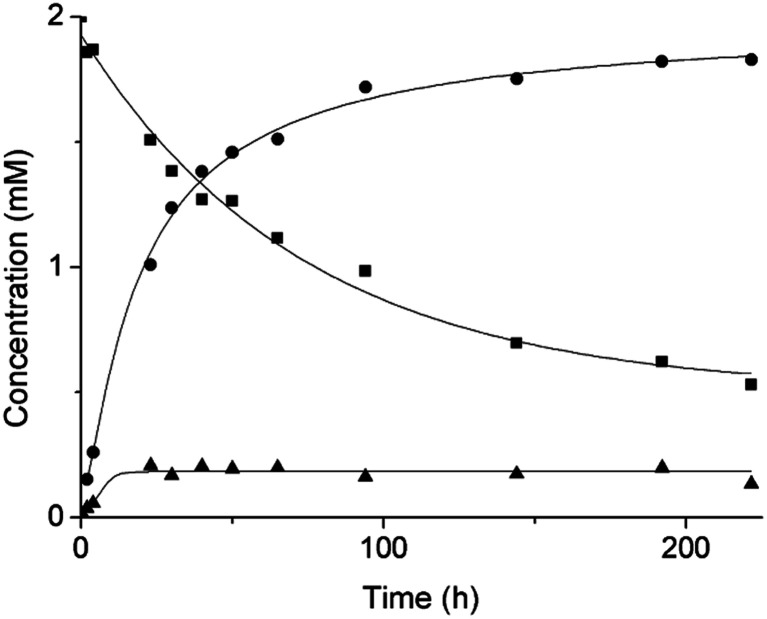
Reaction of 2 mM GG with 2 μmol NU-1000 at 60 °C and pD 7.4: concentration of GG (squares), G (circles) and cG (triangles) as a function of time by ^1^H NMR.

Based on our previous work,^12^ the hydrolytic activity of NU-1000 was initially probed using glycylglycine (GG) as a model substrate. Upon incubation of NU-1000 and GG in D_2_O at pD 7.4 and 60 °C, the hydrolysis of GG dipeptide into two equivalents of glycine (G) was observed by ^1^H NMR spectroscopy, as well as its cyclization to cyclic glycylglycine (cG), which is a side-product of the hydrolytic reaction formed by the intramolecular condensation between the carboxylate and the amino group (Fig. 2). In the presence of NU-1000, the ^1^H NMR spectra showed a steady decrease of the GG signal between 3.82–3.84 ppm and an increase of G resonance at 3.56 ppm and of cG resonance at 4.04 (see Fig. S2 for details†). The concentrations of GG, G and cG were plotted in function of time (Fig. 3) and fitting the data of the concentration of GG to a first order decay function resulted in a rate constant of *k*_obs_ = 1.61 × 10^−6^ s^−1^ at 60 °C and pD 7.4 (Fig. S3†). This corresponds to a half-life (*t*_1/2_) of GG hydrolysis of 120 hours, which represents significant acceleration compared to the non-catalyzed hydrolysis of GG (*t*_1/2_ ≈ 6 years under similar conditions).^32^ Additionally, when using a ten-fold excess of GG (20 μmol) and 2 μmol of NU-1000, ∼90% of the dipeptide was hydrolysed after 432 h of reaction with a rate constant of 1.42 × 10^−6^ s^−1^ (*t*_1/2_ = 136 h), indicating NU-1000 acts as a catalyst for dipeptide hydrolysis (TON = 9) (Fig. S4†). Of note, the reaction products are consistent with the predicted hydrolytic cleavage, likely involving a Zr(iv) Lewis acid activation of the peptide bond that becomes susceptible to the attack of a water molecule, in agreement with our previous systems.^12,33^

Acidity of the reaction medium influenced the reactivity observed, but interestingly, NU-1000 is most active at pD 7.4, making it a suitable catalyst for use at physiological conditions (Fig. S5†). Influence of pD on the hydrolysis of GG was studied in the range from pD 3.4 to 9.4 at 60 °C, and the highest rate was observed at pD 7.4. The decrease of catalyst activity at higher pH values is similar to previously observed with MOF-808, and likely derives from NU-1000's decreased stability at pH > 8 (see discussion below, Fig. S11†). On the other hand, lower reaction rates observed under acidic pH conditions can be explained by the protonation of NH_2_ and COOH terminal groups of GG which disfavors the coordination of the substrate to the electrophilic Zr centers. Therefore, the higher reactivity observed at pD 7.4 most likely results from the best compromise between stability of NU-1000 and protonation state of the substrate.

The reaction temperature affected the activity of NU-1000 in the hydrolysis of GG at pD 7.4, but the rate increase was smaller than observed previously for MOF-808. Upon increasing the temperature from 37 °C to 80 °C, *a* ≈ 11-fold reduction of the half-life from 533 hours at 37 °C to only 46 hours at 80 °C was observed (Fig. S6†). Fitting these data to the Arrhenius equation yields an apparent activation energy (*E*_act_) of 52 kJ mol^−1^ (Fig. S7†). Linear fitting of ln(*k*/*T*) as a function of 1/*T* allows calculating the enthalpy of activation, Δ*H*^‡^ = 49 kJ mol^−1^, and entropy of activation, Δ*S*^‡^ = −221 J mol^−1^ K^−1^, of the reaction (Fig. S8†). The Gibbs energy of activation, Δ*G*^‡^ at physiological temperature (37 °C) was determined to be 118 kJ mol^−1^, *i.e.*, *ca.* 13 kJ mol^−1^ higher than for MOF-808. These numbers reveal a larger entropy penalty for NU-1000 when compared to the experimental value determined for MOF-808 (Δ*S*^‡^ = −116 J mol^−1^ K^−1^), which could indicate a stronger interaction of the substrate with NU-1000. Consequently, upon increasing the temperature, part of this extra energy input would have to be used for substrate and/or products de-coordination, thereby limiting the reaction turnover and resulting in an overall decrease of reaction rate. Even though the reasons for a stronger interaction of GG with NU-1000 than with MOF-808 are not fully understood, the decrease in NU-1000 pore size after the reaction (see discussion below) is consistent with a less labile interaction of GG substrate with the NU-1000 catalyst.

### Hydrolysis of hen egg white lysozyme (HEWL) protein by NU-1000

**Fig. 4:**
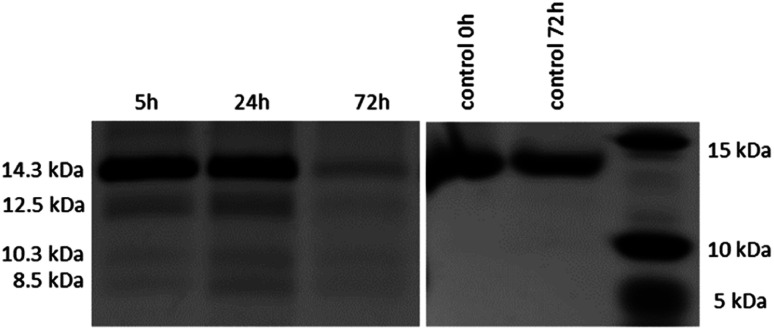
Silver stained SDS-PAGE gel of HEWL incubated at 60 °C and pH 7.0 in the presence of 2 μmol NU-1000 at different time increments (left); control samples of HEWL incubated at 60 °C and pH 7.0 without NU-1000 (right).

After the reactivity of NU-1000 towards peptide bond hydrolysis was established, we turned our attention to explore the desired protease activity. NU-1000 catalytic activity towards protein substrates was evaluated using hen egg white lysozyme (HEWL) protein, a 14.3 kDa globular protein slightly bigger than the largest pore of NU-1000 (HEWL diameter 35 Å *vs.* 31 Å of NU-1000 large pore). HEWL was incubated in the presence of NU-1000 at 60 °C and pH 7.0 in aqueous solution. Elution of protein from the NU-1000 MOF was attempted with several carboxylate buffers as they could replace protein–MOF interactions by interacting with Zr(iv)-centers themselves. Additionally, ammonia 1%, glycine–HCl and guanidine–HCl are used, which are known eluents for proteins by breaking interactions (Fig. S9†).^34,35^ However, HEWL is so strongly adsorbed on NU-1000 that these elution techniques did not show protein or fragment bands on SDS-PAGE gel after Coomassie staining, except the one with ammonia 1%. Elution with ammonia showed the intact protein band, however it also resulted in destruction of the MOF as can be seen on PXRD (Fig. S10†). Since the attempts to recover protein digest from the heterogeneous mixture have proven to be challenging, the heterogeneous MOF–protein mixture was loaded onto a SDS-PAGE gel. As can be seen from Fig. 4, next to the intact protein band at 14.3 kDa new protein fragments at 12.5, 10.3 and 8.5 kDa were observed in the SDS-PAGE gel already after 5 hours of reaction and imaging with silver staining, indicating selective fragmentation of the protein. These bands were not present in control samples incubated under the same conditions but in the absence of MOF catalyst, indicating that the presence of NU-1000 is essential for the hydrolysis to be observed.

Interestingly, protein fragments generated by NU-1000 have comparable molecular weight as the largest fragments observed in the presence of MOF-808 (12.5 *vs.* 12.2 kDa, 10.3 kDa *vs.* 10.7 kDa, and 8.5 kDa *vs.* 8.2 kDa for NU-1000 and MOF-808, respectively, with a 1% error on molecular weight estimated with SDS-PAGE),^12^ indicating the hydrolysis selectivity likely derives from the Zr_6_ cluster and not from the linker structure or the MOF 3D-architecture.

In addition, the molecular weights of these fragments are also strikingly similar to the ones we observed recently upon hydrolysis of HEWL with a Hf(iv)–Wells–Dawson POM complex (Hf–POM),^36^ which has been demonstrated to be an aspartate selective artificial metalloprotease, in full agreement with previous Zr–POM complexes evaluated by us.^21,23^ Given these well-studied precedents and the pivotal role of the Zr_6_ cluster in the selectivity observed, we assumed by analogy that NU-1000 also behaved as an aspartate selective nanozyme. Keeping in mind the intrinsic experimental error in the SDS-PAGE analysis, by extrapolation from our previous works, we suggest that here the HEWL cleavage likely happened at Asp18 affording the heavier fragment (12.5 kDa), Asp52 providing the lightest fragment (8.5 kDa), and a fragment derived from the cleavage at Asp18 and Asp119 (10.3 kDa). This last fragment might be formed by a sequential hydrolysis of the largest fragment formed (12.5 kDa). This fragmentation pattern and the marked influence of Zr_6_ cluster in the reaction selectivity is also consistent with the mechanism for the peptide bond cleavage at aspartate residues proposed for Zr(iv)–POM complexes,^21^ which involves coordination of the peptide bond oxygen to Zr(iv) followed by a direct nucleophilic attack of the carboxylate group in the side chain of Asp on the C-terminal amide carbon.^37^

Despite the striking similarities, analysis of NU-1000 hydrolysis of HEWL by SDS-PAGE gel revealed less fragments than when the hydrolysis was done with MOF-808, and both MOFs intriguingly afford less fragments than our previous Hf(iv)–Wells–Dawson POM catalyst.^36^ More specifically, an additional fragment at 6.4 kDa was observed with MOF-808, while it was absent from the SDS-PAGE of NU-1000 reaction. In addition, while our previous Hf(iv)–POM afforded three fragments of <7 kDa, those were not detected with NU-1000. Given the similarities between NU-1000 and the previous systems, it is plausible that either similar small fragments were not visible on the gel because their concentration is too low or alternatively, they were also produced during the hydrolysis with NU-1000 but remained adsorbed on the MOF. These apparent stronger interaction of lower molecular weight fragments with NU-1000 is consistent with its large mesopores (31 Å), which are almost twice the size of the pores in MOF-808.

### Stability of NU-1000 in the hydrolytic experiments

**Fig. 5:**
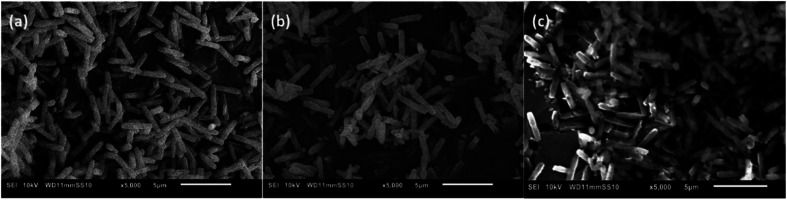
SEM images of (a) NU-1000 as synthesized, (b) after reaction with 2 mM GG, 60 °C, pH 7.0, 24 h, and (c) after reaction with 0.02 mM HEWL, 60 °C, pH 7.0, 24 h.

NU-1000 was synthesized according to literature procedure and its structure has been confirmed by combination of powder X-ray diffraction (PXRD), Fourier-transform infrared (FTIR) and textural analysis (Fig. 5 and S11–13†).^38^ When NU-1000 samples were incubated with GG for 3 days in solutions of pD values ranging from 3.4 to 9.4, an excellent stability between pD 3.4 to 7.4 was observed by PXRD (Fig. S11†). At higher pD, partial loss of stability is evidenced by broadening of the reflections and this can most likely be attributed to linker-node hydrolysis. Loss of stability at alkaline pD is not unexpected as Zr–MOFs in general show structural decomposition in basic aqueous media.^30,39^ Stability at pD 7.4 was further confirmed by FTIR, TGA (Fig. S12 and 13†) and SEM measurements (Fig. 5). TGA analysis also confirmed that the overall temperature stability of NU-1000 was preserved.

NU-1000 acts as a truly heterogeneous catalyst for dipeptide hydrolysis, as the removal of MOF stops the reaction. After incubation of GG with NU-1000 for 3 days (60 °C, pD 7.4), the MOF was removed by centrifugation and the supernatant was allowed to react further at 60 °C. Upon removal of the MOF no further hydrolysis of GG was observed, even after prolonged incubation of 14 days (Fig. S14†), evidencing that the GG hydrolysis only happens in the presence of solid MOF catalyst. Analysis of the supernatant by ICP-OES showed very low concentration of Zr(iv) ions in solution (0.064 ± 0.030 ppm after 24 hours of incubation at 60 °C), indicating hydrolysis was due to the MOF catalyst and not due to soluble Zr(iv) species leached into the solution. These data strongly demonstrate not only the heterogeneous nature of NU-1000 mediated hydrolysis, but also underlines its stability under the reaction conditions.

Due to its heterogeneous nature, NU-1000 can be recycled with minimal loss of activity. To evaluate NU-1000's reusability as a catalyst, the reaction mixture was centrifuged to separate the catalyst after 24 hours of incubation with GG under standard conditions. In order to ensure removal of all residual substrate from the MOF, the samples were stirred with D_2_O overnight before starting the next reaction. Even though this protocol enabled recycling of NU-1000 up to 5 times, after the second run a slight decrease in activity was observed (Fig. S15†). However, PXRD analysis after 3 and 5 cycles did not reveal any structural changes in the catalyst's 3D-framework, demonstrating the material's stability (Fig. S16†). On the other hand, we also observed a decrease in the mass of catalyst recovered after each cycle to losses of handling (≈20% loss of catalyst after five centrifugation runs), which could explain the drop of ≈10% in the third cycle. A decrease of the amount of catalyst resulted in a decreased reaction rate which accounts for the decrease in conversion after 5 hydrolysis cycles (Fig. S17†).

Hydrolysis of proteins is mostly performed in phosphate buffer, which is known for its risk to induce linker-node hydrolysis and consequently loss of structure of MOF, so water at pH 7.0 was used with NU-1000 to make sure its stability is completely preserved.^29,40–43^ Similarly as for GG, PXRD (Fig. S18†), FTIR (Fig. S19†) and SEM (Fig. 5) measurements showed the stability of NU-1000 under conditions used for HEWL hydrolysis after 24 hours. Additionally, TGA analysis confirmed the overall temperature stability of NU-1000 after reaction with HEWL is preserved (Fig. S13†). These results agree with the negligible Zr(iv) leaching observed above, and corroborate the heterogeneous nature of the reaction, since the catalyst is rather stable under all the reaction conditions evaluated.

### Interaction between NU-1000 and peptide and protein substrates

**Fig. 6:**
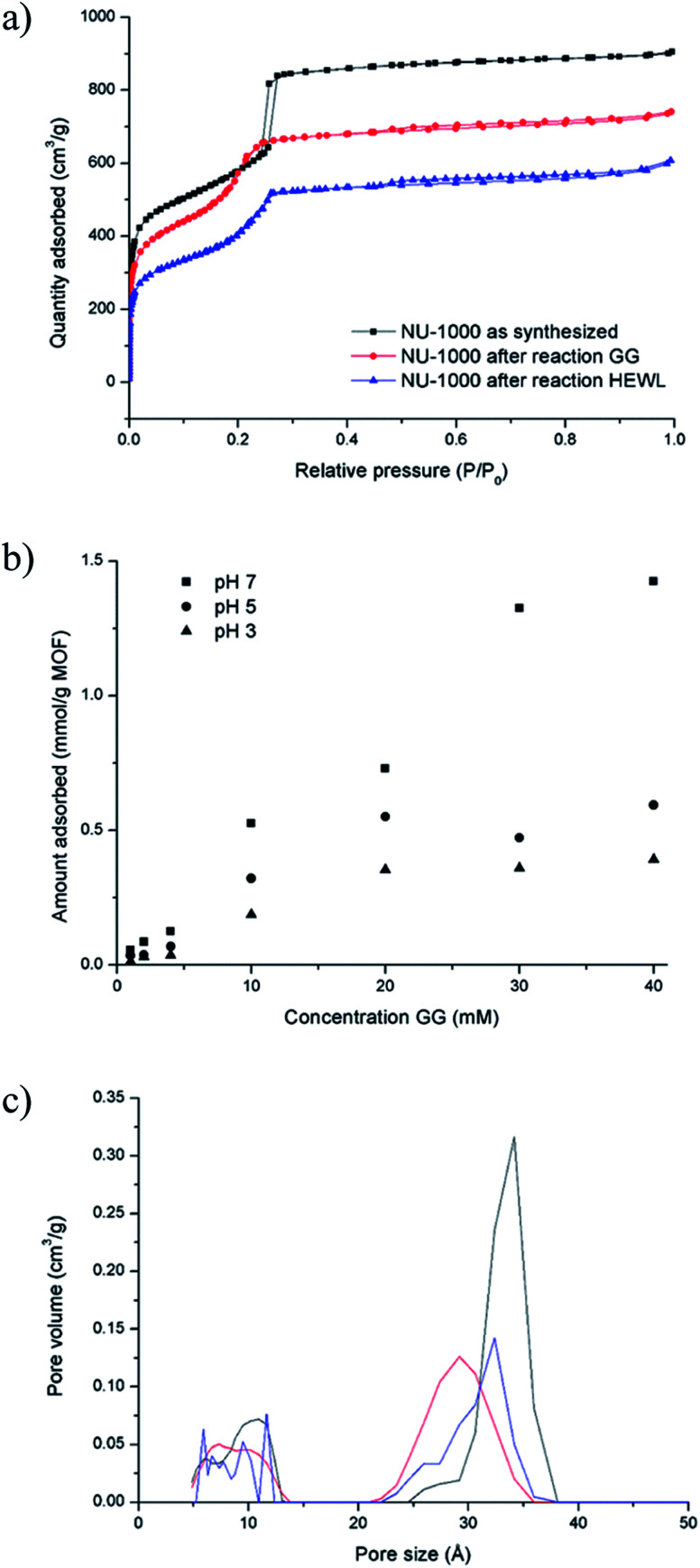
GG and HEWL substrates interact strongly with NU-1000 as shown by physisorption and adsorption experiments: (a) N_2_ isotherm of NU-1000 as synthesized (black), after reaction with 2 mM GG, 60 °C, pH 7.0, 24 h (red), and after reaction with 0.02 mM HEWL, 60 °C, pH 7.0, 24 h (blue). (b) Adsorption of GG on NU-1000 as a function of concentration of GG at pD 7.4, 5.4 and 3.4 (room temperature, 6 h). (c) Pore size distribution of NU-1000 as synthesized (black), after reaction with 2 mM GG, 60 °C, pH 7.0, 24 h (red), and after reaction with 0.02 mM HEWL, 60 °C, pH 7.0, 24 h (blue).

The absence of lower molecular weight fragments and the overall weak intensity of the bands on the SDS-PAGE gel indicated either partial protein adsorption on the NU-1000 surface, or the entrapment into the MOF pores of smaller fragments, and prompted us to investigate this phenomenon in further details. The strong protein adsorption to the MOF matrix was confirmed by incubating HEWL 7 wt% with NU-1000 at room temperature and pH 7, and then analyzing the supernatant by UV-Vis and Tryptophan fluorescence spectroscopy. The results showed that the concentration of HEWL in solution was below the detection limits of these techniques, suggesting its nearly complete adsorption. Additionally, SDS-PAGE analysis of the same supernatant showed no bands of intact protein or fragments after imaging the gel with silver staining, which is known for its high sensitivity, down to ng scale,^44^ indicating an adsorption of over 99% of HEWL on NU-1000 (Fig. S20†). In accordance with the absence of protein in solution, N_2_ physisorption measurements of a NU-1000 sample recovered after the reaction with HEWL showed a reduction of the BET surface area from 2192 to 1612 m^2^ g^−1^, indicating significant adsorption of HEWL (Fig. 6a). Additionally, the loss of organic matter between 50 and 400 °C in TGA suggests the presence of HEWL on the MOF as the protein substrate is degraded at these high temperatures (Fig. S13†).

Adsorption experiments using GG further demonstrate that it enters NU-1000 pores, and illustrate how adsorbed substrates potentially impact the observed reactivity. To follow GG adsorption, increasing concentrations of GG solutions at different pH values (3.0, 5.0 or 7.0) were incubated with NU-1000 at room temperature for 6 hours to ensure that the equilibrium was reached, and then the remaining substrate in solution was quantified by ^1^H NMR (Fig. 6b). In general, less GG was adsorbed when more acidic solutions were used (*i.e.*, adsorption at pH 3.0 < 5.0 < 7.0), which is in line with the slower GG hydrolysis rate observed at more acidic pH values (Fig. S5†). Similarly as evaluated in the hydrolysis of HEWL, possible changes of NU-1000 surface and pores during and after the reaction were probed by performing N_2_ physisorption isotherms with MOF recovered from the GG hydrolysis reactions (Fig. 6a). While only a slight reduction was observed in the BET surface area before and after reaction (2192 and 2161 m^2^ g^−1^ respectively),^45^*a* ≈15% reduction of the largest pore's diameter from 34 to 29 Å was observed after incubation with GG, as indicated by a shift of the second step in the isotherm to lower *P*/*P*_0_ values (Fig. 6c), evidencing the pores are at least partially filled after reaction with GG. Accordingly, small amounts of GG, and G and cG were detected through ^1^H NMR analysis of a digested sample of NU-1000 recovered after reaction (Fig. S21†). These results are in accordance with the linear trend observed in the Arrhenius plot (Fig. S7†), which is characteristic for a reaction without diffusion limitations, and is consistent with the freedom observed of GG to enter and leave the pores in the adsorption experiments.

### On the reactivity of NU-1000 *vs.* MOF-808

**Fig. 7:**
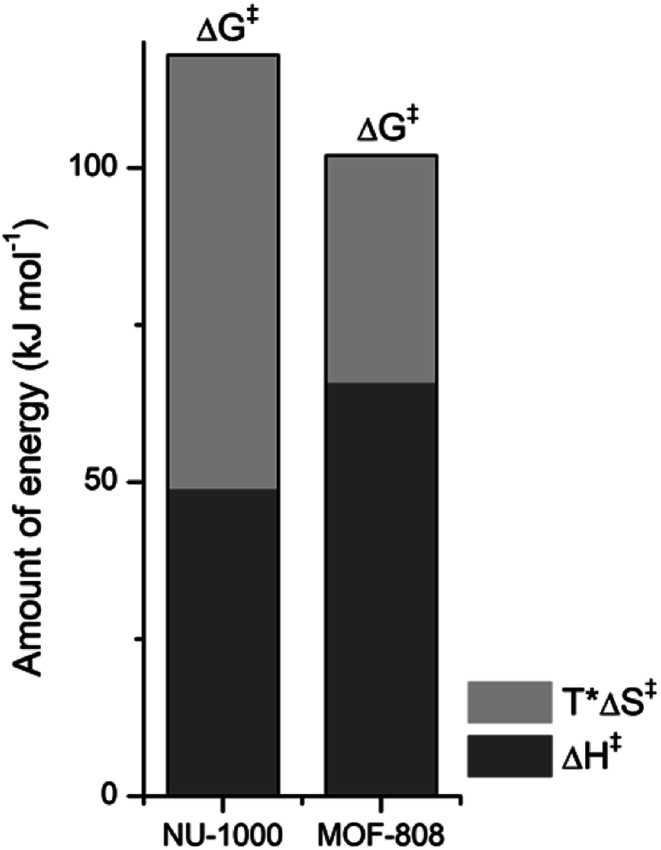
Contribution of enthalpy (dark grey) and entropy (light grey) terms to the Δ*G*^‡^ of peptide bond hydrolysis catalyzed by NU-1000 and MOF-808 (Δ*G*^‡^ = Δ*H*^‡^ − *T*Δ*S*^‡^ with *T* = 37 °C or 310 K, all terms are [kJ mol^−1^]).

NU-1000 and MOF-808 provide similar reactivity patterns in the hydrolysis of GG and HEWL, and both provide faster rates than Zr(iv)-POMs previously investigated,^16,18^ demonstrating the MOF nanozymes are not simple heterogeneous surrogates for homogeneous artificial metallopeptidases but rather more potent catalysts. The similarities in NU-1000 and MOF-808 in response to changes of temperature and acidity of reaction conditions, as well as the cleavage pattern observed with HEWL is a strong evidence that the Zr_6_ cluster is the major factor governing the reactivity, and morphological features derived from their distinct 3D-framework are not governing the reaction outcome. However, NU-1000 and MOF-808 present intriguing differences in the reaction rates that could be related to either molecular features or to macro characteristics like surface area, particle size of each material. Namely, the rate constant for the hydrolysis of GG in the presence of NU-1000 is more than one order of magnitude lower compared to the hydrolysis observed in the presence of MOF-808,^12^ which agrees with the greater experimental value of Δ*G*^‡^ for NU-1000 (118 *vs.* 105 kJ mol^−1^). Clearly, the biggest contribution to this energy difference derives from the Δ*S*^‡^ term, which is twice as large for the NU-1000 in comparison with MOF-808 (−221 *vs.* −116 J mol^−1^ K^−1^), given that the enthalpy of activation of NU-1000 is surprisingly lower than that determined for MOF-808 (49 *vs.* 66 kJ mol^−1^) (Fig. 7). These unexpected activation parameters show that, even though the reaction outcome is governed by the Zr_6_ cluster, its coordination environment completely changes the energy profile of the hydrolysis step.

The difference of connectivity and the material morphology might be at the root of energetic differences observed (Table 1). While NU-1000 is an 8-connected MOF, MOF-808 has only six carboxylate linkers surrounding the Zr_6_ cluster, and therefore contains more catalytic sites, which would explain the faster reactions with MOF-808. It has already been shown that catalytic activity is correlated with the connectivity of Zr_6_ nodes which backs up our hypothesis of the importance of Zr-cluster connectivity.^46,47^ Thus, the lower reaction rates of NU-1000 could simply result from the higher connectivity. However, faster reaction rates have been observed with smaller particle sizes,^48^ and in agreement with this trend NU-1000 particles are larger with a length of 5 μm when compared to MOF-808 particles which are more dispersed in size and have an average diameter around 2 μm.^12^ On the other hand, the reasons behind the difference in Δ*S*^‡^ are difficult to probe experimentally, and our interpretation is that it might reflect the different architectures of MOF-808 and NU-1000 framework. When the GG molecules floating in the bulk solution approach the MOF, it is logical to assume a drop in entropy because the translational movement is restricted by the solid surface both inside and outside the pores. However, the extent of this entropy cost is likely related to the geometry and size of the pores. According to the literature, NU-1000 pores resemble 2D-cylindrical channels, while MOF-808's pores are 3D octahedral and tetrahedral shapes (large and small pores, respectively) (Table 1). In this sense, when GG enters NU-1000 pores it would lose more freedom than when entering MOF-808 pores, resulting in an overall higher Δ*S*^‡^ for the NU-1000 and a subsequent higher Δ*G*^‡^, which would slow the reaction rate. This correlation strongly suggest that even though the connectivity has a major effect in the observed reactivity, other structural aspects such as shape and dimension of the pores can have an effect over activation parameters as important as the Lewis acidity of Zr_6_ nodes and/or the stereoelectronic features of the linkers, being highly relevant for the reaction kinetics. Therefore, not only the nature of MOF building blocks but also the overall architecture need to be considered in future designs, as they have a large impact on the catalytic activity by affecting the Δ*S*^‡^ term.

Comparison of cluster and particle characteristics of NU-1000 and MOF-808 and their combined effect on half-life for hydrolysis of GG

**Table:** 

Characteristic	NU-1000		MOF-808
Connectivity Zr_6_ cluster	8	>	6
Particle size (μm)	5	>	2
Half-life (h)	120	>	0.72

**Table:** 

Pore size (Å)	31	18.4
	12	7–10
Pore shape	2D channel	3D
	Hexagonal (large)	Octahedral (large)
	Triangular (small)	Tetrahedral (small)

## Conclusion

In summary, we provided detailed analysis of kinetic, thermodynamic and structural data for NU-1000 MOF mediated hydrolysis of peptide bond. Comparing these data with the structural and thermodynamic parameters obtained for MOF-808 nanozyme, allowed us to identify key parameters that influence hydrolytic reaction catalyzed by Zr_6_ based MOFs. More specifically, a rather unexpected large contribution of the Δ*S*^‡^ term to the Δ*G*^‡^ of the hydrolysis reactions suggests that not only connectivity but also 3D-features of the MOF architecture can have a strong influence on the kinetics of the reaction. Such finding differs to the common approach in literature of modulating the electronics/structure of the ligand or the composition of metal nodes to optimize MOF catalytic activity. Therefore, it provides a new angle for the design of future MOF catalysts. In addition, the protease activity of NU-1000 towards a HEWL protein under physiological pH afforded selective cleavage at only 3 peptide bonds. This showcases the potential of Zr–MOFs as selective heterogeneous catalysts suitable for protein hydrolysis in modern biotechnological and proteomic applications. Further studies probing the interplay between electronic and morphological features of MOFs in the kinetics of peptide hydrolysis, and refining the protease activity of MOF-based nanozymes are ongoing in our lab and will be reported in due course.

## Author contributions

A. L., S. S., J. M. and C. S. performed the experiments reported in this work. F. A., D. D. V., and T. N. P. V helped with the design and interpretation of the experiments. The manuscript was written through contributions of all authors. All authors have given approval to the final version of the manuscript.

## Conflicts of interest

There are no conflicts to declare.

## Supplementary Material

SC-011-D0SC02136A-s001
