# Supplementary material for: Interplay between structural parameters and reactivity of Zr6-based MOFs as artificial proteases
Source: Chem Sci. 2020 May 22;11(26):6662–9. doi: 10.1039/d0sc02136a (PMC8159359; doi:10.1039/d0sc02136a)
Supplement: SC-011-D0SC02136A-s001 [file SC-011-D0SC02136A-s001.pdf]

Electronic Supplementary Material (ESI) for Chemical Science.
This journal is © The Royal Society of Chemistry 2020

Supporting Information

Interplay between structural parameters and reactivity of Zr_6-based MOFs as artificial proteases

Alexandra Loosen,[a] Francisco de Azambuja,[a] Simon Smolders,[b] Jens Moons,[a] Charlotte Simms,[a] Dirk De Vos,[b] and Tatjana N. Parac-Vogt*[a]

* E-mail: tatjana.vogt@kuleuven.be

[a] Department of Chemistry, KU Leuven, Celestijnenlaan 200F, Leuven, Belgium

[b] Department Microbial and Molecular Systems, KU Leuven, Celestijnenlaan 200F, 3001 Leuven, Belgium

Table of Contents

Experimental Procedures 3

Synthesis of tetraethyl-4,4′,4″,4‴-(pyrene-1,3,6,8-tetrayl)tetrabenzoate (1) 4

Synthesis H_4TBAPy linker: saponification of (1) 4

Synthesis of NU-1000[1] 4

Results and Discussion 5

Figure S2. ^{1}H NMR spectra recorded at various time increments for the reaction of 2 mM GG with 2 µmol NU-1000 at 60 °C and pD 7.4........ 5

Figure S3. First order decay fit of ln[GG] as a function of time for the reaction of 2 mM GG with 2 µmol NU-1000 at 60 °C and pD 7.4........ 5

Figure S4. Reaction of 20 mM GG with 2 µmol NU-1000 at 60 °C and pD 7.4. a) Concentration of GG as a function of time by ^{1}H NMR. b) First order decay fit of ln[GG] as a function of time for the reaction of 20 mM GG with 2 µmol NU-1000 at 60 °C and pD 7.4........ 5

Figure S5. Influence of pD on conversion of 2 mM GG with 2 µmol NU-1000, 60 °C, 3 days. 6

Figure S6. Influence of temperature on reaction rate of hydrolysis of 2 mM GG with 2 µmol NU-1000, pH 7.0, as a function of reciprocal temperature. 6

Figure S7. Arrhenius plot of reaction rate as a function of reciprocal temperature for reaction of 2 mM GG and 2 µmol NU-1000, pH 7.0........ 6

Figure S8. ln(k/T) plotted as a function of reciprocal temperature for reaction of 2 mM GG with 2 µmol NU-1000, pH 7.0. ..7

Figure S9. Coomassie stained SDS-PAGE gel of sample of HEWL incubated at 60 °C and pH 7.0, in the presence of 2 µmol NU-1000 under different elution techniques........ 7

Figure S10. PXRD pattern of NU-1000 as synthesized (black) and after reaction with 0.02 mM HEWL in water, 60 °C, pH 7.0, 3 days and 3 days of elution with 1 % ammonia hydroxide (red). 7

Figure S11. PXRD patterns of NU-1000 as synthesized and after incubation with 2 mM GG at 60 °C for 3 days at different pD values. 8

Figure S12. FTIR spectrum of (a) NU-1000 as synthesized, (b) after reaction with 2 mM GG, 60 °C, pH 7.0, 24 h and (c) GG........ 8

Figure S13. Thermal gravimetric analysis of NU-1000 as synthesized (black), after reaction with 2 mM GG, 60 °C, pH 7.0, 24 h (red) and after reaction with 0.02 mM HEWL, 60 °C, pH 7.0, 24 h (blue). 9

Figure S14. % Conversion of GG in function of time before (black) and after (red) removal of NU-1000........ 9

Figure S15. Recycling experiment of NU-1000 after solvent exchange with D_2O; five reaction cycles with 6 µmol NU-1000 and 2 mM GG, 60 °C, pD 7.4, 24 h in 3 mL D_2O. 9

Figure S16. PXRD patterns of NU-1000 as synthesized (black) and after three (red) and five (blue) reaction cycles with 2 mM GG, 60 °C, pH 7.0, 24 h and solvent exchange with D_2O overnight. 10

Figure S17. Influence of amount of MOF on reaction rate for hydrolysis of 2 mM GG by NU-1000, 60 °C, pH 7.0. 10

Figure S18. PXRD pattern of NU-1000 as synthesized and after reaction with 0.02 mM HEWL in water, 60 °C, pH 7.0, 24 h. 11

Figure S19. FTIR spectrum of (a) NU-1000 as synthesized, (b) after reaction with 0.02 mM HEWL, 60 °C, pH 7.0, 24 h and (c) HEWL. 11

Figure S20. Silver stained SDS-PAGE gel of supernatant after centrifugation of sample of HEWL incubated at 60 °C and pH 7.0, in the absence and presence of 2 µmol NU-1000 at different time increments. 12

Figure S21. ^1H NMR spectrum of 2 µmol of NU-1000 in DMSO-*d*$_6$, digested with D_2SO_4 and diluted 2 times (after reaction with 2 mM GG, 60 °C, pH 7.0, 24 h). Substrate peaks in aliphatic region and 0.12 µM TMSPA-*d*$_4$ as internal standard at 0 ppm. 12

References **12**

Experimental Procedures

Synthesis procedures. H_4TBAPy linker was synthesized according to published methods via standard Suzuki-Miyaura reaction between 1,3,6,8-tetrabromopyrene and 4-ethoxycarbonylphenylboronic acid with some modifications. The ester is saponified by excess of base to get full conversion of the ester and after acidification the carboxylate linker is obtained with an overall yield of 47 % (Figure S1). The Zr-MOF NU-1000 was synthesized according to literature.[1] NU-1000 is prepared via an upscaled solvothermal method in which zirconyl chloride octahydrate is mixed with the H_4TBAPy linker in *N,N*-dimethylformamide with benzoic acid as modulator.[1] The modulator is removed postsynthetically by washing with a 37 % HCl solution. After thermal activation, the desired product is characterized with powder X-ray diffraction, fourier-transform infrared spectroscopy, scanning electron microscopy, thermogravimetric analysis and N_2 physisorption. ^{1}H NMR spectra are obtained by acidic digestion of 1 mg NU-1000 with 5 drops of D_2SO_4 in 1 mL of DMSO-d_6.

Before hydrolysis experiments, NU-1000 was activated at 120 °C for 20 h to evacuate adsorbed molecules from the pores.

Hydrolysis studies of dipeptides. 800 µL of D_2O was mixed with 4.32 mg NU-1000, corresponding to 2 µmol of Zr_6 clusters. 200 µL of a 10 mM Gly-Gly (GG) solution was added and pD was adjusted to 7.4 with NaOD. Of note, pD was measured in a conventional pH meter observing the relationship pD = pH_{read} + 0.41 to account for the isotopic composition of the solvent.[2] Reactions were done in individual vials at 60 °C and at certain time points, the solution of a vial was centrifuged at 14000 rpm for 2 x 10 min, after which the supernatant solution was sampled for analysis. Temperature dependence studies followed the same procedure with reactions performed at 37, 50, 60, 70 and 80 °C over time. For pD dependence, reactions were carried out in a pD range between 3.4 and 9.4 with intervals of one, each after 3 days of reaction at 60 °C. All the reactions were followed with ^{1}H NMR spectroscopy and 3-(trimethylsilyl)propionic-2,2,3,3-d_4 acid sodium salt (TMSPA-d_4) was added to the supernatant before measurements. Reaction rates were determined by fitting a first-order decay function to the data.

Hydrolysis of proteins. 800 µL of H_2O was added to 2 µmol NU-1000 and mixed with 200 µL of hen egg white lysozyme (HEWL) solution (0.1 mM); pH was adjusted to 7.0. Reactions were done at 60 °C and 15 µL samples, collected at different time points, each from a separate reaction vial, were mixed with 5 µL sample buffer. Samples were run on 18 % Laemmli gels in a OmniPAGE electrophoretic cell at 200 V for 1.5 h. Page Ruler unstained low range protein ladder was used as a standard. Silver staining was used for imaging the gel and analysis was done with ImageLab.[3]

Elution studies of HEWL. 800 µL of H_2O was added to 2 µmol NU-1000 and mixed with 200 µL of hen egg white lysozyme (HEWL) solution (0.1 mM); pH was adjusted to 7.0. Reactions were done at 60 °C for 3 days and centrifuged. 1 mL of elution buffer was added to the precipitate and stirred for 3 days at room temperature. 15 µL samples were mixed with 5 µL sample buffer. Samples were run on 18 % Laemmli gels in a OmniPAGE electrophoretic cell at 200 V for 1.5 h. Page Ruler unstained low range protein ladder was used as a standard. Coomassie staining was used for imaging the gel and analysis was done with ImageLab.[3]

Recycling experiment. Reactions were conducted the same way as described above for the hydrolysis of dipeptides. After 24 h, the catalyst was recovered by centrifugation, and was regenerated by stirring overnight in D_2O, followed by centrifugation at 14000 rpm for 10 min. This cycle was repeated in total five times, each for 24 h with 2 mM GG and after each cycle, centrifugation and stirring overnight in D_2O was performed before starting the next cycle. Conversion rates were calculated from ^{1}H-NMR analysis of the supernatant whereas stability was determined with powder X-ray diffraction and inductively coupled plasma optical emission spectrometry.

Adsorption studies of HEWL. 800 µL of H_2O was mixed with 2 µmol NU-1000. 200 µL of a 0.1 mM hen egg white lysozyme (HEWL) solution was added and pH was adjusted to 7.0 with NaOH. Reactions were done in individual vials at room temperature and at certain time points, the solution of a vial was centrifuged at 14000 rpm for 2 x 10 min, after which the supernatant solution was sampled for analysis with SDS-PAGE, UV-Vis and Tryptophan fluorescence spectroscopy.

Adsorption studies of GG. A stock solution of 40 mM GG was prepared and dilutions were made by adding the appropriate amount of GG to 2 µmol NU-1000 in D_2O resulting in samples of 1, 2, 4, 10, 20, 30 and 40 mM GG. Concentration series were measured at pD 7.4, 5.4 and 3.4 by adjusting pD with NaOD and DCl. After 6 hours of stirring at room temperature, samples were centrifuged and ^{1}H NMR analysis of the supernatant was performed to determine the substrate uptake.

Instrumentation. ^{1}H NMR spectra were recorded with a Bruker Avance 400 and 600 spectrometer in deuterated solvents and with 0.1 M TMSPA-d_4 as an internal reference. Spectra were analyzed using Topspin software.[4] Powder X-ray diffraction (PXRD) patterns were collected on a Malvern PANalytical Empyrean diffractometer (in transmission mode) over a 1.3 – 45° 2θ range, using a PIXcel3D solid state detector and Cu anode (Cu $K_{\alpha 1}$: 1.5406 Å; Cu $K_{\alpha 2}$: 1.5444 Å). Fourier-transform infrared spectra (FTIR) were recorded on a Bruker Vertex 70 spectrometer and analyzed with the Bruker OPUS software (version 7.5).[5] The solid samples were measured directly, without sample preparation, using the attenuated total reflectance module (Platinum ATR). Scanning electron microscopy (SEM) micrographs were recorded using a JEOL-6010LV SEM after depositing a palladium/gold layer on the samples with a JEOL JFC-1300 autofine coater under Ar plasma. N_2 physisorption isotherms were measured on a Micromeritics 3Flex surface analyzer at -196 °C. Prior to measurements, samples were evacuated at 120 °C under vacuum for 12 h. Surface areas were calculated using the multi-point BET method applied to the isotherm adsorption branch taking into account the Rouquerol consistency criteria and the micropore volume was calculated at P/P_0 = 0.5.[6] Thermal gravimetric analyses (TGA) were performed under air atmosphere on a NETZSCH STA 449 F3 Jupiter® thermal analyser with a heating rate of 4 °C per minute. Inductively coupled plasma optical emission spectrometry (ICP-OES) was measured on a PerkinElmer optical emission spectrometry Optima 8300 instrument. UV-Vis measurements were performed on a

Varian Cary 5000 UV-VIS-NIR spectrometer. Tryptophan fluorescence was measured on a Edinburgh Instruments FLS980 Spectrofluorimeter.

1,3,6,8-tetrabromopyrene (97 %, TCI), 4-ethoxycarbonylphenylboronic acid (TCI), K_3PO_4 (Sigma Aldrich), $Pd(PPh_3)_4$ (Sigma Aldrich), dioxane (99.5 %, Acros Organics), acetone (Fisher Scientific), chloroform (Fisher Scientific), methanol (Fisher Scientific), KOH (Acros Organics), HCl (37 %, ChemLab), $ZrOCl_2.8H_2O$ (98 %, Alfa Aesar), benzoic acid (Sigma Aldrich), *N,N*-dimethylformamide (Fischer Scientific), D_2SO_4 (Sigma Aldrich), NaOD (40 wt%, Sigma Aldrich), TMSPA-d_4 (Sigma Aldich), DMSO-d_6 (Sigma Aldrich). All chemicals were commercially obtained and used without further purification.

Synthesis of tetraethyl-4,4′,4″,4‴-(pyrene-1,3,6,8-tetrayl)tetrabenzoate (1)

4 g of 1,3,6,8-tetrabromopyrene (7.72 mmol), 6.6 g of 4-ethoxycarbonylphenylboronic acid (34.02 mmol), 13.2 g of K_3PO_4 (62.18 mmol) and 0.6 g of $Pd(PPh_3)_4$ (0.52 mmol) are added together in 216 mL dioxane under N_2 atmosphere under continuous stirring. The suspension is stirred for 48-72 h at 90 °C under N_2 atmosphere. The solution becomes more and more yellow and turns black when the reaction is complete. 160 mL of H_2O is added and the mixture is cooled down. The solution is filtered and the yellow precipitate is washed twice with 80 mL of H_2O and twice with 160 mL of acetone. The precipitate is then dissolved in 240 mL of boiling chloroform. The volume is reduced under vacuum by half and subsequently 240 mL of methanol is added. After 30 minutes a yellow precipitate is recovered by centrifugation and dried at 70 °C under vacuum for 12 h (yield: 3.17 g; 52 %).

^{1}H NMR (400 MHz, $CDCl_3$): δ (ppm) = 8.25-8.23 (d, 8H, phenyl H), 8.15 (s, 4H, pyrene H), 8.01 (s, 2H, pyrene H), 7.76-7.74 (d, 8H, phenyl H), 4.48-4.43 (q, 8H, CH_3-C**H**$_2$-O), 1.55-1.44 (t, 12H, C**H**$_3$-CH_2-O).

Synthesis H_4TBAPy linker: saponification of (1)

3.17 g of (1) (3.99 mmol) is added in 160 mL dioxane. 220 mL of a KOH solution (0.4 M) is added to the suspension which is then refluxed for 24 h. The precipitate is filtered while hot to remove the greyish residue. After cooling down and upon adding 30 mL of HCl 37 % a yellow precipitate is formed. This precipitate is centrifuged and washed 3 times with 100 mL of H_2O. The product is dried overnight at 100 °C under vacuum and then for 3 h at 80 °C, also under vacuum. (yield: 2.45 g; 90 %)

^{1}H NMR (400 MHz, DMSO-d_6): δ (ppm) = 13.11 (br, 4H, COOH), 8.20-8.15 (s+d, 12H, pyrene+phenyl H) 8.08 (s, 2H, pyrene), 7.87-7.85 (d, 8H, phenyl H).

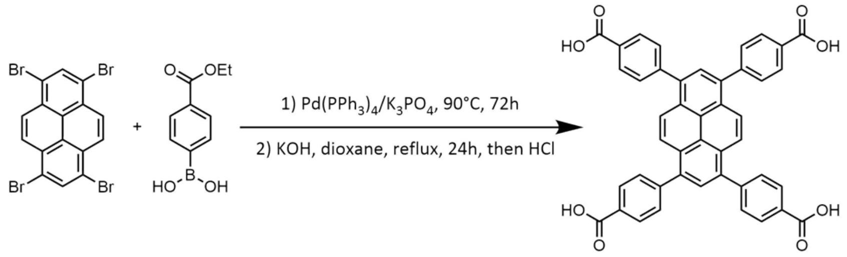

Figure S1. Synthesis of H_4TBAPy.

Synthesis of NU-1000[1]

1.94 g of ZrOCl2.8H2O (6.02 mmol) and 54 g of benzoic acid (0.44 mol) are added together in 120 mL of N,N-dimethylformamide (DMF) in a 500 mL VWR pressure plus+ Schott bottle, sonicated until fully dissolved and put in an oven at 100 °C for 1 h. 0.800 g of H4TBAPy (1.17 mmol) is added in 40 mL of DMF in a 100 mL VWR pressure plus+ Schott bottle and sonicated till a suspension is obtained. This suspension is also put in an oven at 100 °C for 1 h and a clear solution is obtained. Both solutions are mixed together in a 500 mL VWR pressure plus+ Schott bottle and placed in an oven at 120 °C for 16 h. After cooling down, the precipitate is centrifuged and washed 3 times with 45 mL of DMF, each time soaking for 2 h. After centrifugation, the precipitate is added to 260 mL of DMF and 10 mL of HCl (8 M) is added dropwise while shaking gently. This mixture is placed in a 100 °C oven overnight. After cooling down, the precipitate is washed 3 times with 45 mL of DMF for 2 h and 3 times with 45 mL of acetone, each time soaking for 12 h. The product is dried at 80 °C under vacuum for 1 h (yield: 1.06 g; 84 %). The structure of the Zr-NU-1000 is confirmed with PXRD, FTIR, SEM and N2 physisorption. 1H NMR is obtained by sonication of 1 mg of Zr-NU-1000 in 5 drops of D2SO4 and dissolving in 1 mL DMSO-d6.

1H NMR (400 MHz, DMSO-d6): δ (ppm) = 8.09-8.05 (m, 12H, pyrene+phenyl), 7.95 (s, 2H, pyrene), 7.76-7.74 (d, 8H, phenyl).

Results and Discussion

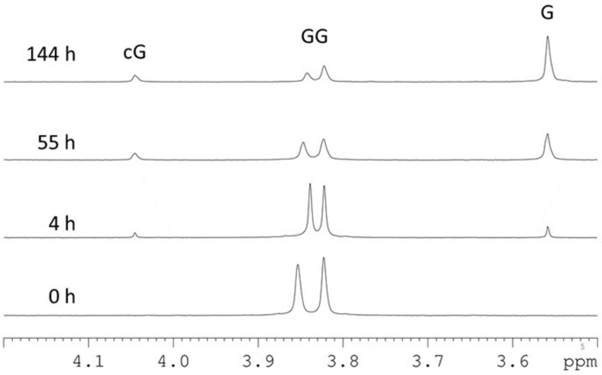

Figure S2. ^{1}H NMR spectra recorded at various time increments for the reaction of 2 mM GG with 2 μmol NU-1000 at 60 °C and pD 7.4.

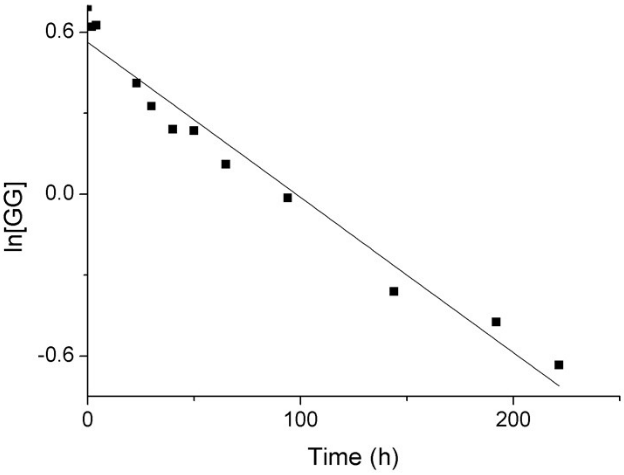

Figure S3. First order decay fit of ln[GG] as a function of time for the reaction of 2 mM GG with 2 μmol NU-1000 at 60 °C and pD 7.4.

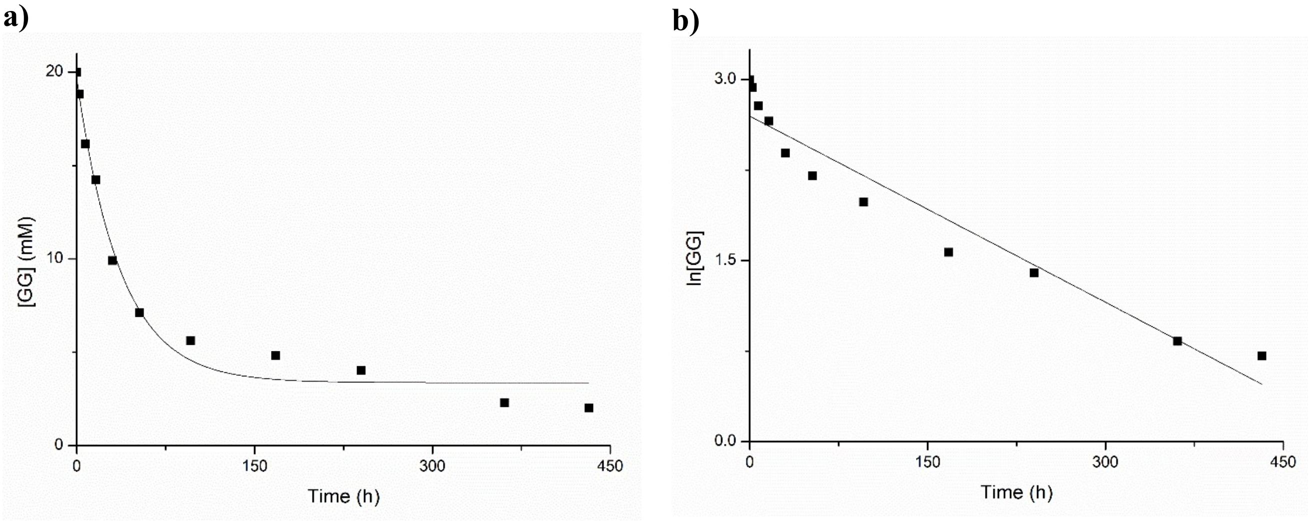

Figure S4. Reaction of 20 mM GG with 2 μmol NU-1000 at 60 °C and pD 7.4. a) Concentration of GG as a function of time by ^{1}H NMR. b) First order decay fit of ln[GG] as a function of time for the reaction of 20 mM GG with 2 μmol NU-1000 at 60 °C and pD 7.4.

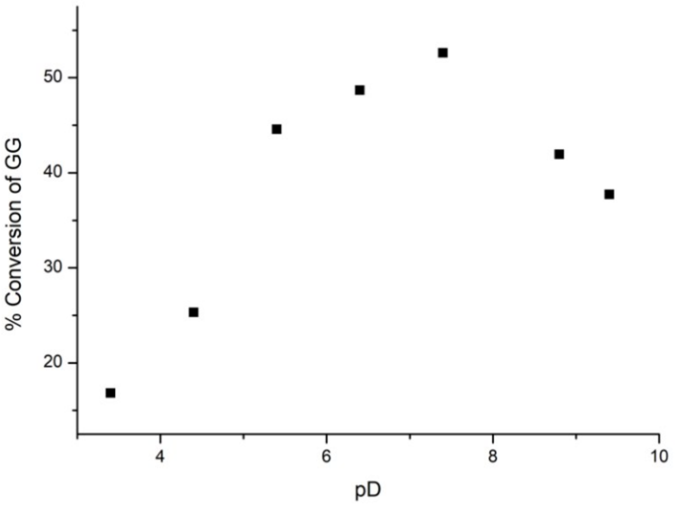

Figure S5. Influence of pD on conversion of 2 mM GG with 2 μmol NU-1000, 60 °C, 3 days.

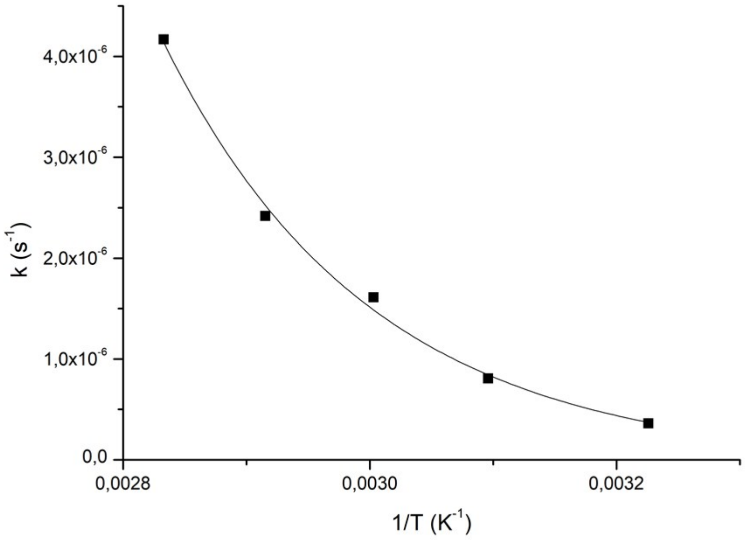

Figure S6. Influence of temperature on reaction rate of hydrolysis of 2 mM GG with 2 μmol NU-1000, pH 7.0, as a function of reciprocal temperature.

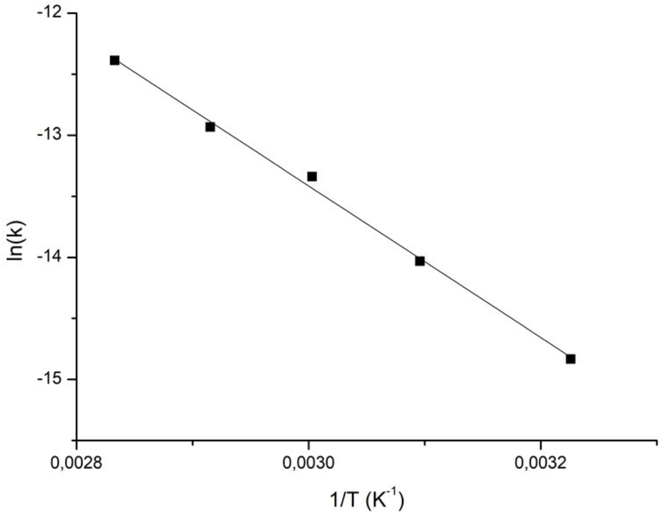

Figure S7. Arrhenius plot of reaction rate as a function of reciprocal temperature for reaction of 2 mM GG and 2 μmol NU-1000, pH 7.0.

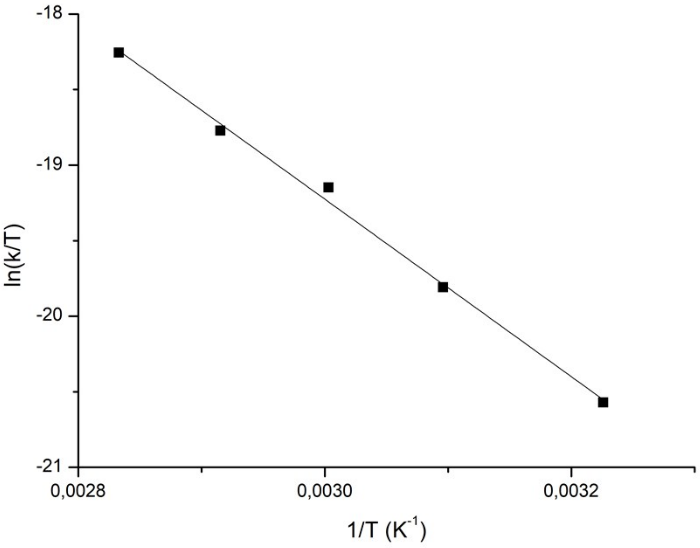

Figure S8. ln(k/T) plotted as a function of reciprocal temperature for reaction of 2 mM GG with 2 µmol NU-1000, pH 7.0.

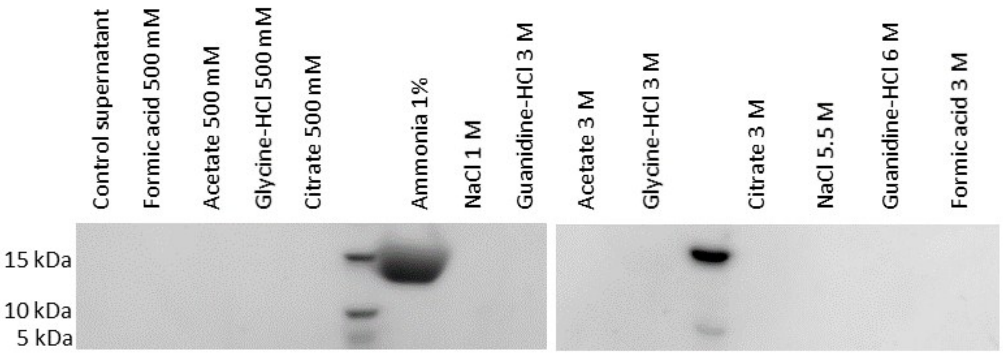

Figure S9. Coomassie stained SDS-PAGE gel of sample of HEWL incubated at 60 °C and pH 7.0, in the presence of 2 µmol NU-1000 under different elution techniques.

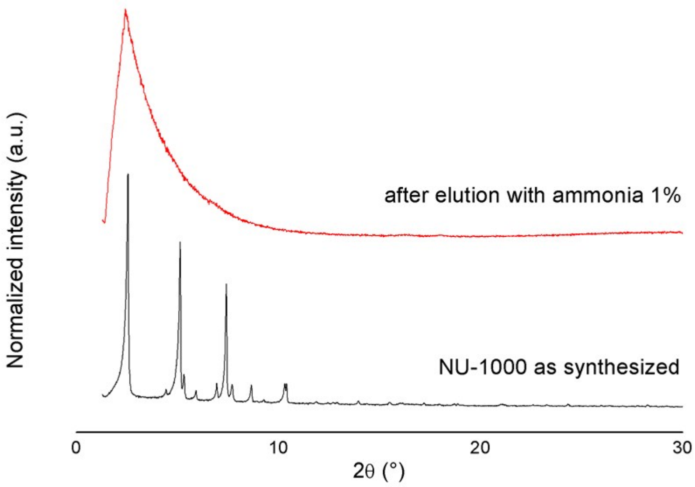

Figure S10. PXRD pattern of NU-1000 as synthesized (black) and after reaction with 0.02 mM HEWL in water, 60 °C, pH 7.0, 3 days and 3 days of elution with 1 % ammonia hydroxide (red).

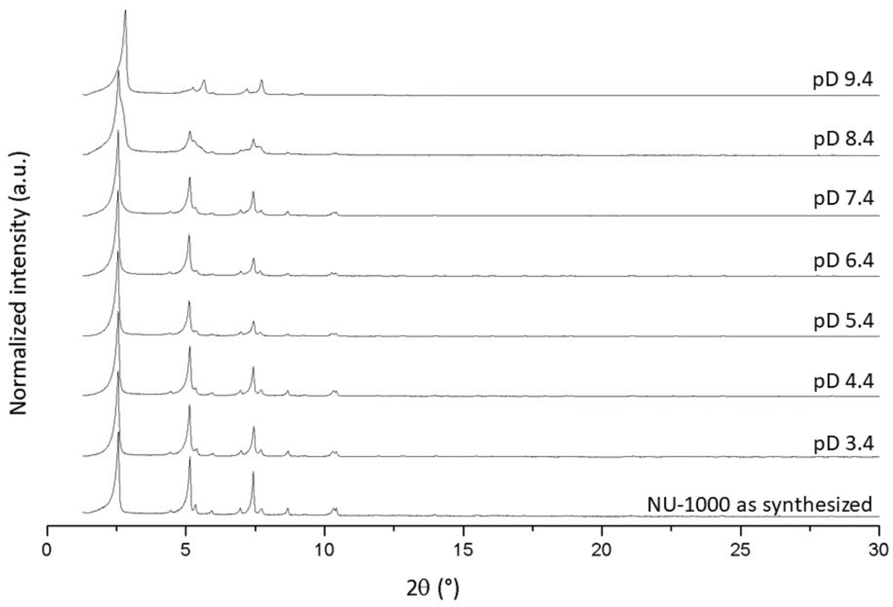

Figure S11. PXRD patterns of NU-1000 as synthesized and after incubation with 2 mM GG at 60 °C for 3 days at different pD values.

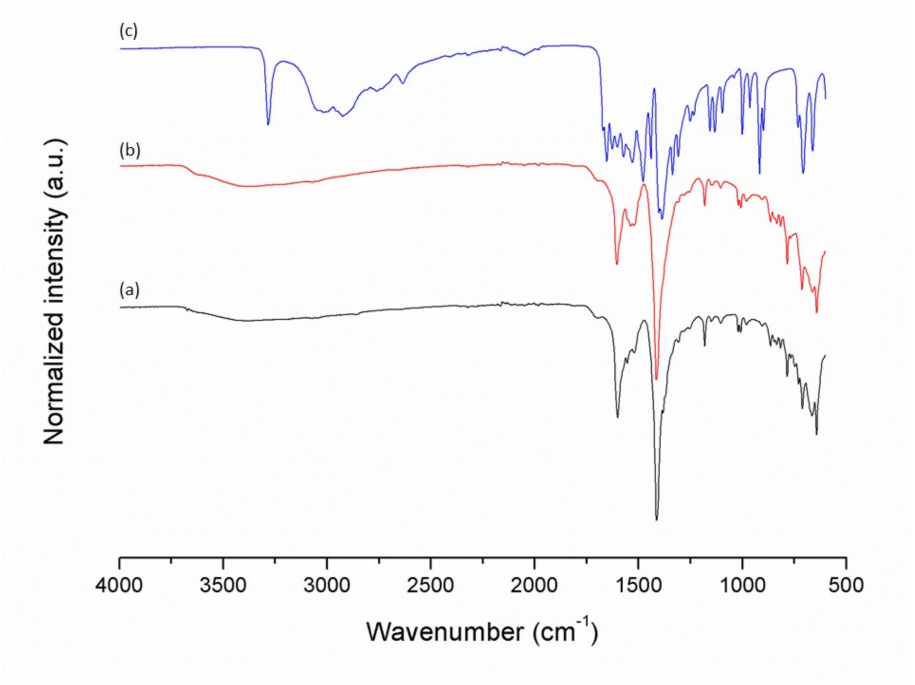

Figure S12. FTIR spectrum of (a) NU-1000 as synthesized, (b) after reaction with 2 mM GG, 60 °C, pH 7.0, 24 h and (c) GG.

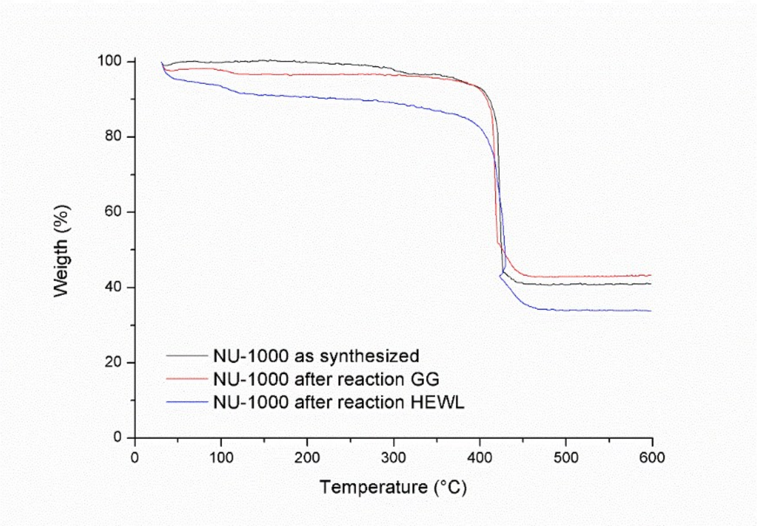

Figure S13. Thermal gravimetric analysis of NU-1000 as synthesized (black), after reaction with 2 mM GG, 60 °C, pH 7.0, 24 h (red) and after reaction with 0.02 mM HEWL, 60 °C, pH 7.0, 24 h (blue).

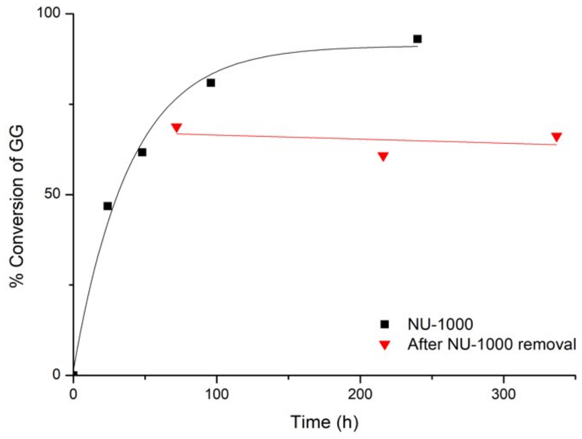

Figure S14. % Conversion of GG in function of time before (black) and after (red) removal of NU-1000.

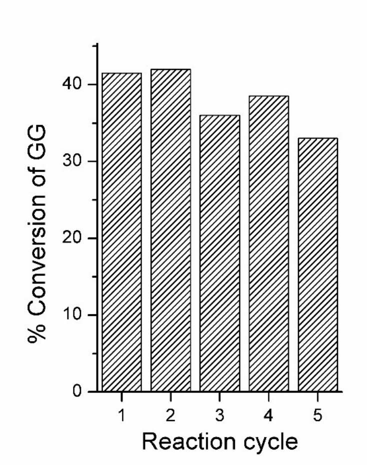

Figure S15. Recycling experiment of NU-1000 after solvent exchange with D_2O; five reaction cycles with 6 μmol NU-1000 and 2 mM GG, 60 °C, pD 7.4, 24 h in 3 mL D_2O.

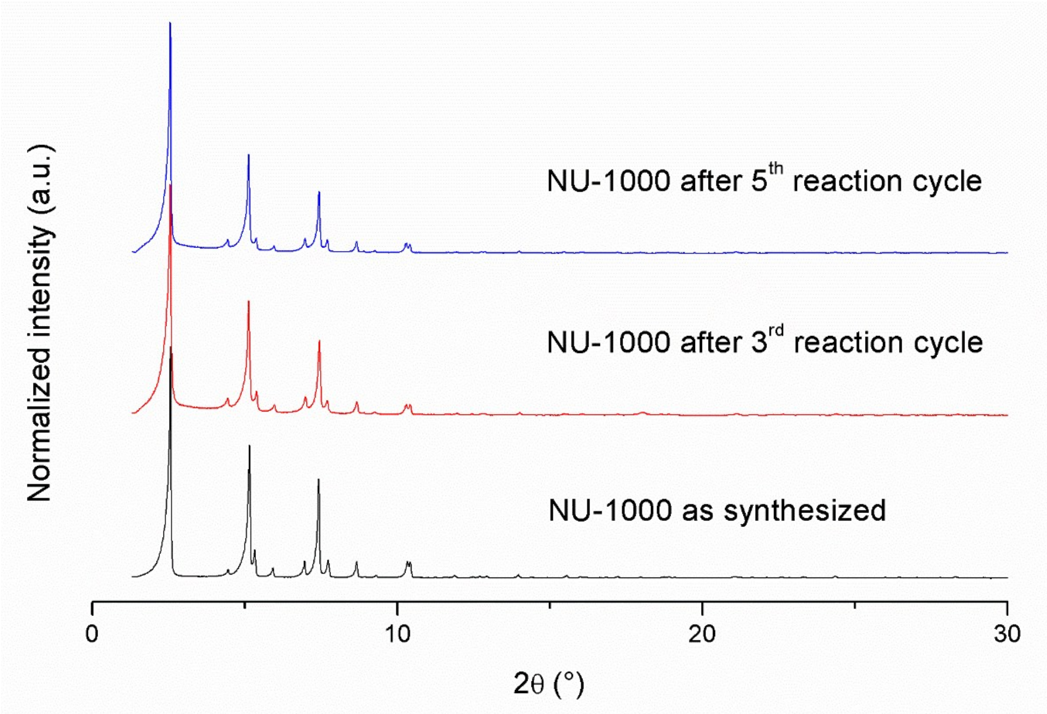

Figure S16. PXRD patterns of NU-1000 as synthesized (black) and after three (red) and five (blue) reaction cycles with 2 mM GG, 60 °C, pH 7.0, 24 h and solvent exchange with D_2O overnight.

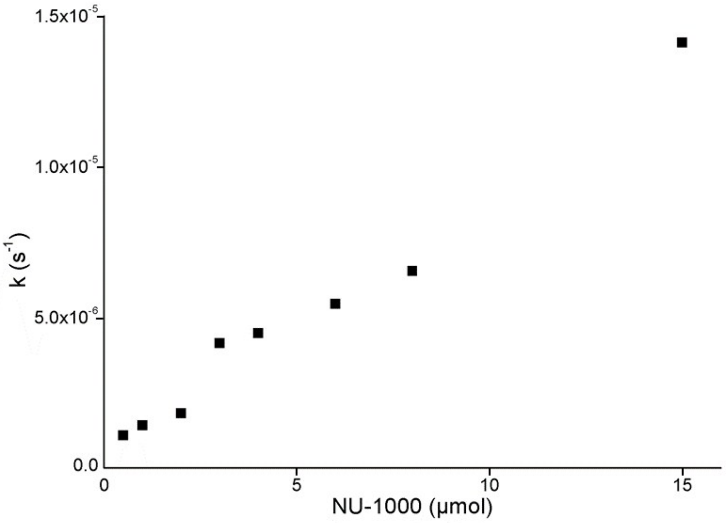

Figure S17. Influence of amount of MOF on reaction rate for hydrolysis of 2 mM GG by NU-1000, 60 °C, pH 7.0.

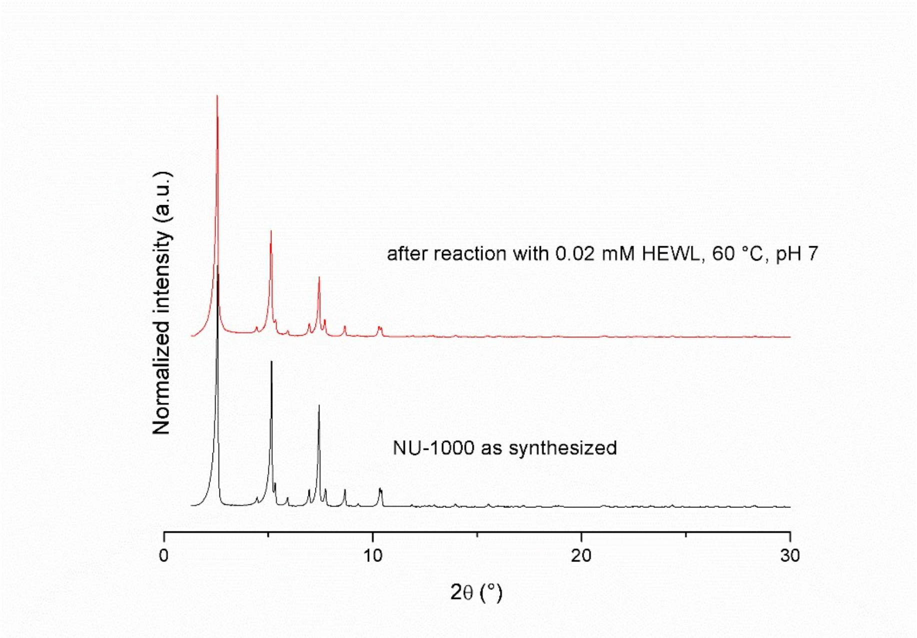

Figure S18. PXRD pattern of NU-1000 as synthesized and after reaction with 0.02 mM HEWL in water, 60 °C, pH 7.0, 24 h.

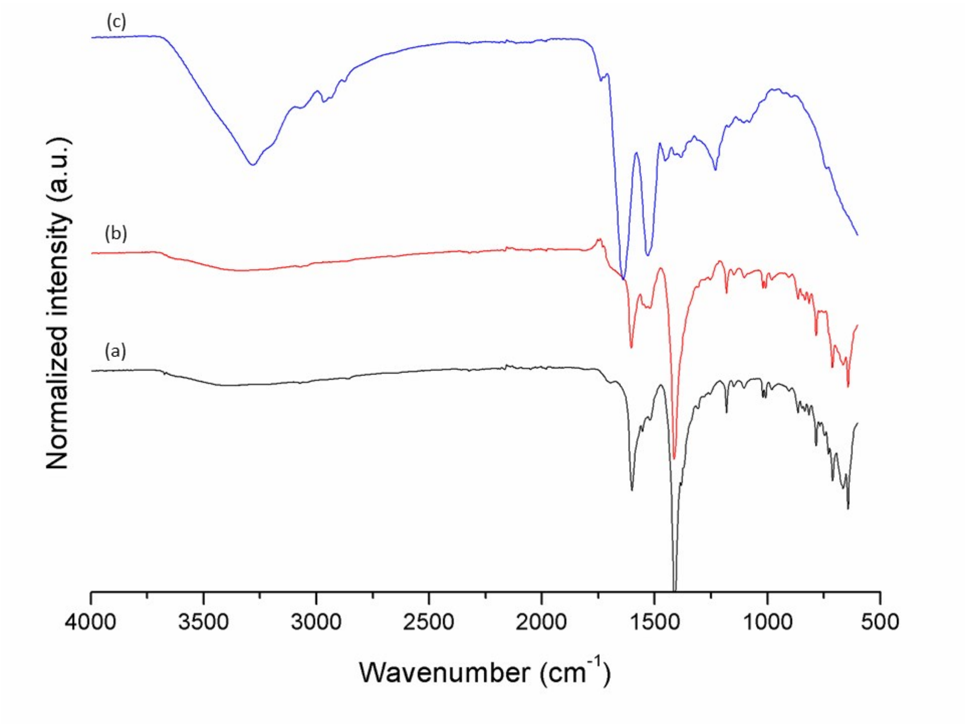

Figure S19. FTIR spectrum of (a) NU-1000 as synthesized, (b) after reaction with 0.02 mM HEWL, 60 °C, pH 7.0, 24 h and (c) HEWL.

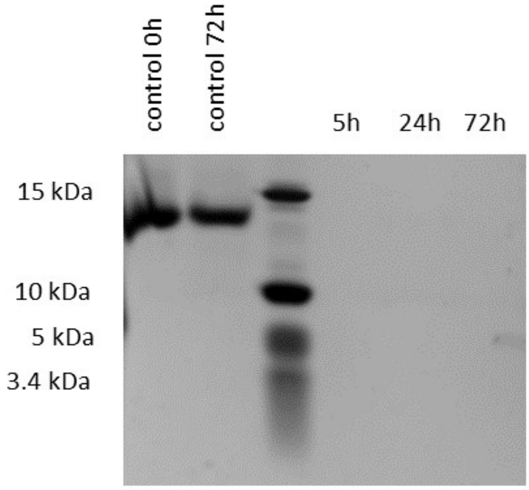

Figure S20. Silver stained SDS-PAGE gel of supernatant after centrifugation of sample of HEWL incubated at 60 °C and pH 7.0, in the absence and presence of 2 µmol NU-1000 at different time increments.

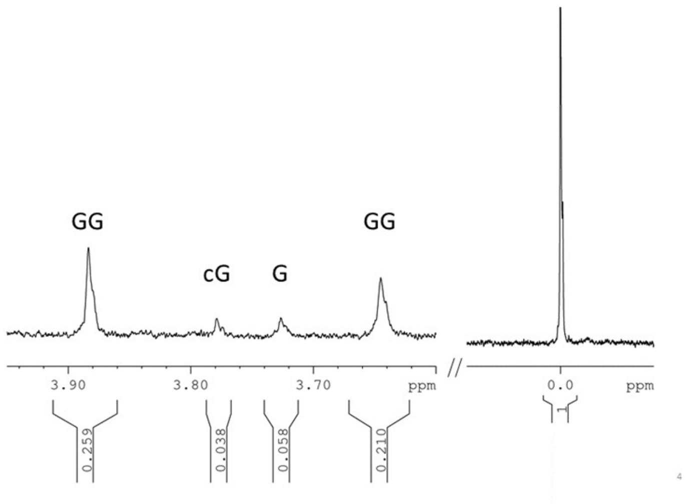

Figure S21. ^{1}H NMR spectrum of 2 µmol of NU-1000 in DMSO-d_6, digested with D_2SO_4 and diluted 2 times (after reaction with 2 mM GG, 60 °C, pH 7.0, 24 h). Substrate peaks in aliphatic region and 0.12 µM TMSPA-d_4 as internal standard at 0 ppm.

References

1. Wang, T. C.; Vermeulen, N. A.; Kim, I. S.; Martinson, A. B.; Stoddart, J. F.; Hupp, J. T.; Farha, O. K., "Scalable synthesis and post-modification of a mesoporous metal-organic framework called NU-1000." *Nature Protocol* **2016,** *11*, 149-62.
2. Glasoe, P. K.; Long, F. A., "Use of glass electrodes to measure acidities in deuterium oxide." *J. Phys. Chem.* **1960,** *64*, 188-190.
3. Laboratories, B.-R. *Image Lab*, 3.0; 2010.
4. Bruker *TopSpin*, 3.6.1; 2018.
5. Bruker *OPUS Spectroscopy Software*, 7.5.
6. Rouquerol, J.; Rouquerol, F.; Sing, K. S. W., *Absorption by Powders and Porous Solids*. Academic Press: London, 1999.
